# Potential Hypoglycaemic and Antiobesity Effects of* Senna italica* Leaf Acetone Extract

**DOI:** 10.1155/2018/5101656

**Published:** 2018-02-19

**Authors:** R. O. Malematja, V. P. Bagla, I. Njanje, V. Mbazima, K. W. Poopedi, L. Mampuru, M. P. Mokgotho

**Affiliations:** Department of Biochemistry, Microbiology and Biotechnology, Faculty of Science and Agriculture, University of Limpopo, Turfloop Campus, Private Bag X1106, Sovenga, Limpopo 0727, South Africa

## Abstract

**Background:**

Type II diabetes is on the rise while obesity is one of the strongest risk factors of type II diabetes. The search for a drug for type II that can equally mitigate obesity related complication is desired.

**Methods:**

The acetone leaf extract of* Senna italica* was evaluated for its cytotoxic, antiglycation, and lipolytic effect, glucose uptake, and GLUT4 translocation and expression using published methods, while that for adipogenesis and protein expression levels of obesity related adipokines was assessed using adipogenesis assay and mouse adipokine proteome profiler kit, respectively. The possible mechanism of glucose uptake was assessed through the inhibition of PI3K pathway.

**Results:**

The extract had no adverse effect on 3T3-L1 cell viability (CC50 > 1000 *μ*g/ml). High antiglycation effect was attained at 10 mg/ml, while at 25–200 *μ*g/ml it showed no significant increase in adipogenesis and lipolysis. The extract at 100 *μ*g/ml was shown to decrease the expression levels of various adipokines and minimal glucose uptake at 50–100 *μ*g/ml with a nonsignificant antagonistic effect when used in combination with insulin. GLUT4 translocation and expression were attained at 50–100 *μ*g/ml with an increase in GLUT4 expression when in combination with insulin.

**Conclusion:**

The acetone leaf extract of* S. italica* stimulates glucose uptake through the PI3K-dependent pathway and can serve as a source of therapeutic agent for the downregulation of obesity-associated adipokines in obesity and antiglycation agents.

## 1. Introduction

Diabetes mellitus (DM) is a group of metabolic disorders characterised by a hyperglycaemic condition resulting from defects in insulin secretion, insulin action, or both [1]. The condition occurs in three forms, namely, types I and II and gestational diabetes. Type II diabetes is on the rise as a global health problem. Several pathogenic processes are involved in the development of diabetes. These range from autoimmune destruction of the *β*-cells of the pancreas with consequent insulin deficiency, to abnormalities that result in resistance to insulin action in major target organs such as the liver, muscle, and adipose tissue [2, 3].

Obesity on the other hand is associated with type II diabetes, due to its link with induced insulin resistance [4]. The incidence of insulin resistance is on the rise worldwide, hand-in-hand with the rise in obesity [5]. However, the relationship between obesity and insulin resistance is not completely understood. Diabetes is associated with serious long-term complications which can lead to chronic morbidities and mortality [6]. A nonenzymatic process known as glycation on the other hand becomes accelerated as a result of persistent elevated plasma glucose levels which occur in diabetic patients.

The use of natural medicines appears to offer better means of managing diseases at lower cost [7, 8].* Senna italica*, with synonyms* Cassia italica* and* Acacia obovata* belongs to the family of Fabaceae.* Senna* species have been of extreme interest in phytochemical and pharmacological research due to their excellent medicinal values. They are well known in folk medicine for their laxative and purgative effect. Besides, they have been found to exhibit anti-inflammatory, antioxidant, hypoglycemic, antiplasmodial, larvicidal, antimutagenic, and anticancer activities [9]. Furthermore, antioxidant constituents in plants were shown to play a critical role in the management of diabetes mellitus [10]. This study was therefore aimed at evaluating the potential effect of* S. italica* on GLUT4 translocation, expression, and adipogenesis in 3T3-L1 preadipocyte cells.

## 2. Materials and Methods

### 2.1. Plant Collection and Preparation

The leaves of* S. italica* were collected from Zebediela region, Limpopo Province, South Africa. The plant material with Voucher specimen number UNIN11129 is deposited at the Larry Leach Herbarium of the University of Limpopo. Dried plant material was ground into a fine powder using a commercial electric blender and stored in air-tight bottles. Twenty grams of leaf material was extracted with 200 ml acetone (Sigma-Aldrich, Germany). The mixture was shaken overnight at room temperature using a Series 25 shaking incubator machine (New Brunswick Scientific Co. Inc., USA) set at 200 rpm. The extraction procedure was repeated three times in order to extract as much components as possible. The extract was filtered using Whatman no. 1 filter paper and dried under a stream of cold air. The dried extract material was stored in preweighed vials for further use.

### 2.2. Determination of Anti-Glycation Activity

Antiglycation activity of* S. italica* acetone leaf extract was determined using bovine serum albumin (BSA) assay according to [11] with minor modifications. Briefly, BSA (1 mg/ml) was incubated with fructose (500 mM) in phosphate buffer (PB) containing 0.02% of sodium azide at pH 7.4 for 30 days at 37°C. The incubation was conducted in the presence and absence of the plant extract (1.25–10 mg/ml). Incubations without plant extract served as a negative control and arbutin was used as a standard inhibitor. Corresponding blanks were prepared in the absence of fructose. The reaction was terminated by adding 10 *μ*l of 100% trichloroacetic acid (TCA) and centrifuged (13,000 rpm) at 4°C for 4 min. The supernatant was then discarded and the pellet was redissolved in 500 *μ*l of alkaline phosphate buffer saline (PBS) (Thermo-Fisher Scientific, USA) at pH 10. Fluorescence was measured using Glomax Microtiter Plate Reader (Promega, USA).

### 2.3. Cytotoxicity Assay

The effect of* S. italica* acetone leaf extract on viability of 3T3-L1 preadipocyte cells was evaluated using the 3-(4,5-dimethylthiazol-2-yl)-2,5-diphenyltetrazolium bromide (MTT) assay [12]. The cells were plated at a density of 5 × 10^3^ cells/well in Dulbecco's minimum-eagle's medium (DMEM) (Thermo-Fisher Scientific, USA) supplemented with 10% foetal bovine serum, FBS (Thermo-Fisher Scientific, USA). The plated cells were treated with various concentrations (25–800 *μ*g/ml) of* S. italica* acetone leaf extract in 96-well microtiter plates (Whitehead Scientific, RSA) and incubated at 37°C in a humidified incubator (5% CO_2_) for 24 and 48 h. Thereafter, the treatment media was removed. Two mg/ml of MTT (Sigma-Aldrich, Germany) solution was added to each well and incubated for 4 h. The MTT solution was removed from the wells and 100 *μ*l of dimethyl sulfoxide (DMSO) was added to each well and agitated to dissolve the formazan deposit. The absorbance was recorded using a Glomax Microtiter Plate Reader (Promega, USA) at 560 nm.

### 2.4. Differentiation of 3T3-L1 Preadipocytes

3T3-L1 preadipocytes were differentiated as described by [13]. The cells were treated with adipocyte differentiation media containing 10% FBS (Thermo-Fisher Scientific), 1 *μ*M dexamethasone (DEX) (Sigma-Aldrich, Germany), 0.5 mM 3-isobutyl-1-methylxanthine (Sigma-Aldrich, Germany), and 1 *μ*g/ml insulin (Sigma-Aldrich, Germany) for 3 days. Thereafter, the differentiation media was substituted with adipocyte maintenance media (DMEM, 10% FBS, and 1 *μ*g/ml insulin) for a further 2 days. On day 5 the adipocyte maintenance medium was substituted with DMEM containing 10% FBS and cells were cultured until day 8. On day 8 the cells were fully differentiated to mature adipocytes.

### 2.5. Lipolysis Assay

Cells were seeded in a 96-well plate (20,000 cells/ml) and differentiated. After differentiation, the cells were washed twice with lipolysis wash buffer (Sigma-Aldrich, Germany) and then treated with various concentrations (25–200 *μ*g/ml) of* S. italica* acetone leaf extract. Hundred micromolar isoproterenol was used as a positive control and untreated cells as negative control. Plates were incubated at 37°C in a 5% CO_2_ atmosphere for 3 h. Fifty microliters of the treatment from each well was aspirated and transferred to a new 96-well assay plate and mixed with lipolysis assay reaction mix (Sigma-Aldrich, Germany) and further incubated for 30 min at room temperature. To measure the amount of glycerol released, absorbance was recorded using Glomax Microtiter Plate Reader (Promega, USA) at 560 nm.

### 2.6. Adipogenesis

Cells were seeded in a 96-well plate (20,000 cells/ml) and incubated to form a confluent monolayer. The cells were thereafter treated with different concentrations of* S. italica* acetone leaf extract for 48 h. Adipocyte differentiation medium (ADM) was used as a positive control and untreated cells as negative control. After 48 h treatment, the medium was removed and cells were washed once with PBS. Hundred microliters of lipid extraction buffer was added in each well, plate sealed, and incubated for 30 min at 90°C. The plate was cooled and agitated to homogenise the solution. Fifty microliters of each treatment was added to a new 96-well assay plate and 2 *μ*l of lipase solution (Sigma-Aldrich, Germany) was added to each well and incubated for 10 min at room temperature. Thereafter, 50 *μ*l of adipogenesis master mix was added to each well and further incubated for 30 min. In order to measure the amount of accumulated triglycerides, absorbance was recorded using Glomax Microtiter Plate Reader (Promega, USA) at 560 nm.

### 2.7. Mouse Adipokine Array Dot Blot

Cells were grown in 75 cm^2^ flasks (White Scientific, RSA) and differentiated. After differentiation, cells were treated for 24 h with* S. italica* acetone leaf extract (100 *μ*g/ml) with or without 100 ng/ml lipopolysaccharide (LPS). Lipopolysaccharide alone was used as a positive control and untreated cells used as a negative control. Cells were washed with PBS and lysed with lysis buffer (1% Igepal CA-630, 20 Mm Tris-HCl (pH 8.8), 137 mM NaCl, 10% glycerol, and complete EDTA-free protease inhibitor cocktail) using tissue lyser (Retsch., Germany) for 5 min and centrifuged at 13,000 rpm for 10 min at 4°C (Whitehead Scientific, RSA). The expression of adipokines in the cells was determined using the mouse adipokine profiler assay kit according to the manufacturer's instructions (R&D Systems). Briefly, membranes were blocked for 1 h on a rocking platform shaker. Protein samples (500 *μ*g) were prepared and 15 *μ*l of reconstituted mouse adipokine detection antibody cocktail was added to each prepared sample, mixed, and incubated at room temperature for 1 h. Sample/antibody mixtures were then added and incubated overnight at 2–8°C on a rocking platform shaker. After incubation, membranes were washed with wash buffer and 2 ml of streptavidin-HRP in Array Buffer 6 (1 : 2000) was added into each membrane and incubated for 30 min at room temperature on a rocking platform shaker. Each membrane was then washed and removed from its wash container allowing excess wash buffer to drain from the membrane. The membranes were detected using ChemiDoc XRS Image Analyser (Bio-Rad, USA).

### 2.8. Glucose Uptake Assay

Glucose uptake assay was measured using the method of [14] with slight modification. Briefly, 3T3-L1 preadipocytes cells were cultured and differentiated, after which they were untreated/pretreated with 50 *μ*M of LY294-002 for 30 min to inhibit PI3K. The cells were further incubated in Krebs-Ringer bicarbonate buffer (110 mM NaCl, 4.4 mM KCl, 1.45 mM KH_2_PO_4_, 1.2 MgCl_2_, 2.3 CaCl_2_, 4.8 mM NaHCO_3_, 10 mM HEPPES, and 0.3% BSA) containing 50 *μ*g/ml and 100 *μ*g/ml* S. italica* acetone extract with and without insulin for 4 h. Insulin (1 *μ*M) was used as a positive control and untreated cells as a negative control. Glucose oxidase (ITinder-GOD/PAP) (KAT Medical, RSA) was thereafter added and incubated for 20 min at 37°C. The absorbance was recorded using Glomax Microtiter Plate reader (Promega, USA) at 560 nm.

### 2.9. GLUT4 Translocation Assay

Cells were seeded in a corning cell bind 24-well plate (20,000 cells/ml) and differentiated. After differentiation, the cells were treated with 50 *μ*g/ml and 100 *μ*g/ml of* S. italica* acetone leaf extract in the presence or absence of 1 *μ*M insulin for 4 h. Untreated cells were used as negative control and 1 *μ*M insulin as positive control. After treatment, cells were washed and blocked with 0.5% bovine serum albumin (BSA) and incubated with rabbit polyclonal GLUT4 primary antibody (1 : 200) (Santa Cruz Biotechnology, USA) for 30 min at 37°C. After 30 min, cells were washed and further incubated with goat anti-rabbit IgG FITC (conjugated) (Santa Cruz Biotechnology, USA) for 30 min in the dark at room temperature. Cells were then washed with PBS and images were captured by fluorescence microscope (Nikon, Japan) at 40x magnification.

### 2.10. Western Blotting

3T3-L1 preadipocytes were seeded at a density of 2 × 10^4^ cells/ml in a 25 cm^2^ cell culture flask (Whitehead Scientific, RSA). Cells were then treated for 24 h (5% CO_2_, 37°C) with 50 *μ*g/ml and 100 *μ*g/ml of* S. italica* acetone leaf extract in the presence and absence of 1 *μ*M insulin. Insulin (1 *μ*M) was used as a positive control. After treatment, cells were then washed with PBS and lysed with lysis buffer (1% N-P40, 50 mM Tris-HCl (pH 8.8), 150 mM NaCl, 0.5% sodium deoxycholate, 0.1% sodium dodecyl sulphate (SDS), and complete EDTA-free protease inhibitor cocktail) using tissue lyser (Retsch., Germany) for 5 min and centrifuged at 13,000 rpm for 10 min at 4°C (Whitehead Scientific, RSA). The supernatant was transferred into a clean 2 ml tube and protein concentration was determined using the detergent compatible (DC) protein assay (Bio-Rad, USA). This was followed by separating 30 *μ*g of the total protein using 12% SDS-PAGE (Bio-Rad, USA) before transferring the gels onto the membrane. Membranes were immersed in transfer buffer (Bio-Rad, USA) and 100% methanol for 2 min. The gel was then transferred to the membranes using trans-blot turbo transfer system (Bio-Rad, USA). After transferring, the bands were visualised using ponceau stain (Sigma-Aldrich, Germany). Then membrane was washed and blocked with 5% skimmed milk for 90 min followed by incubation with GLUT4 mouse monoclonal antibody (Cell Signaling, RSA) 1 : 500 dilutions at 4°C overnight. After incubation the membrane was washed with TBST followed by incubation with goat anti-rabbit antibodies (Santa Cruz Biotechnology, USA) 1 : 4000 dilutions for 90 min at room temperature. The membrane was washed and substrate was added. Antigen antibody complex was visualised by photo-detection using the Syne-Gene Image Analyser (Bio-Rad, USA).

### 2.11. Statistical Analysis

GraphPad Instat Software was used for statistical analysis. The results were obtained from three independent replicate experiments and expressed as means ± SEM. The statistical significance of the results was tested using one-way ANOVA employing the Tukey-Kramer Multiple Comparisons Test. Statistical significance was considered at *p* < 0.05.

## 3. Results

### 3.1. Antiglycation Effect of* S. italica* Acetone Leaf Extract

**Figure 1:**
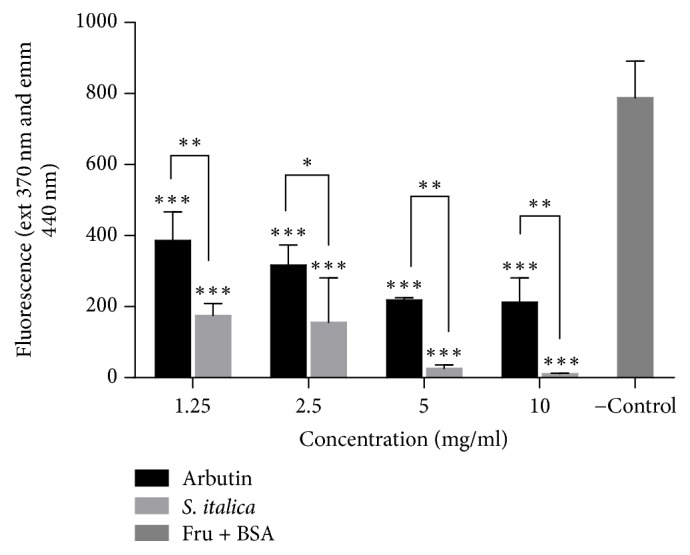
Effect of* S. italica* on fluorescence intensity of glycated BSA. Fructose and BSA were incubated with various concentrations of* S. italica* 1.25–10 mg/ml. The experiment was carried out for 30 days. Arbutin was used as a positive control and fructose + BSA as a negative control. Data represents the ± SEM of 3 independent experiments, ^*∗∗∗*^*p* < 0.001, ^*∗∗*^*p* < 0.01, and ^*∗*^*p* < 0.05.

The ability of the extract and arbutin to inhibit glycation was comparable to the control and was significantly high at all concentrations (*p* < 0.001). Antiglycation was concentration-dependent for both arbutin and the plant extract. The extract exhibited a significantly high glycation ability compared to arbutin (Figure 1).

### 3.2. Effect of* S. italica* Acetone Leaf Extract on Viability of 3T3-L1

**Figure 2:**
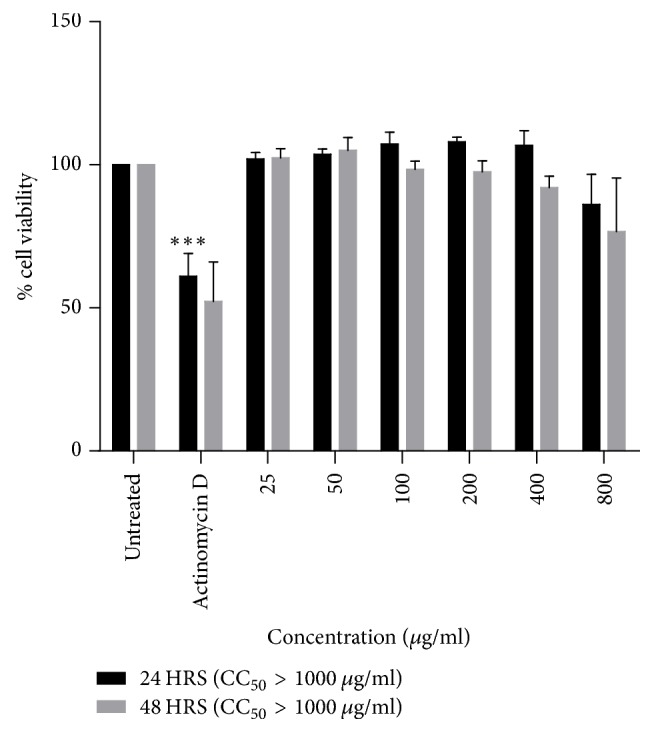
The effect of* S. italica* acetone leaf extract on 3T3-L1 preadipocytes cells treated with various concentrations (25–800 *μ*g/ml). The experiment was carried out for 24 and 48 h using MTT assay. Untreated cells and Actinomycin D were used as negative and positive controls, respectively. Data represents the ± SEM of 3 independent replicate experiments, ^*∗∗∗*^*p* < 0.001.

The plant extract at both 24 and 48 h of treatment was not cytotoxic on 3T3-L1 cells at the concentrations used in the study. However, prolonged incubation with increased concentration of the extract affected cell viability. The CC_50_ values of the extract at 24 and 48 h were >1000 *μ*g/ml, Figure 2.

### 3.3. Effect of* S. italica* Acetone Leaf Extract on Lipolysis

**Figure 3:**
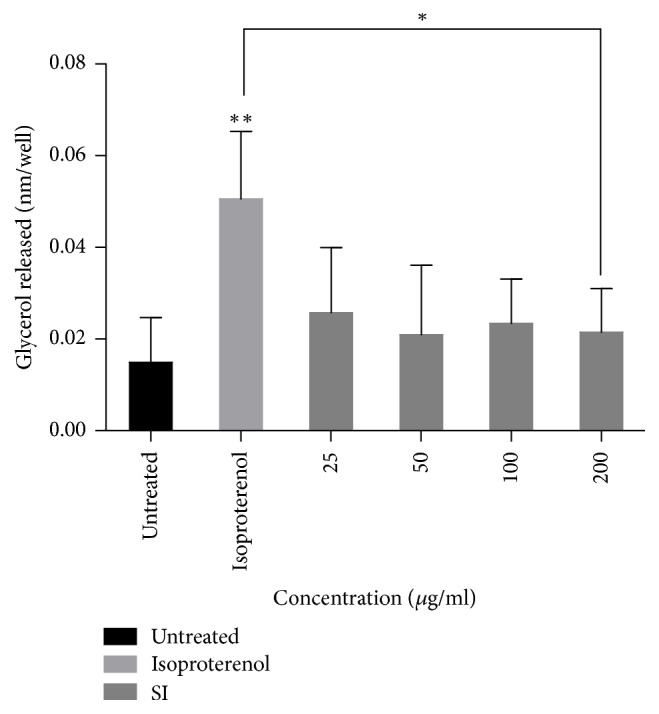
Effect of* S. italica* acetone leaf extract on lipolysis. Differentiated 3T3-L1 cells were treated with various concentrations (25–200 *μ*g/ml) of* S. italica* extract. Isoproterenol was used as a positive control and untreated cells as negative control. The experiment was carried out for 3 h. Data represents the ± SEM of 3 independent replicate experiments, ^*∗∗*^*p* < 0.01 and ^*∗*^*p* < 0.05.

The results show that lipolytic activity of the extract decreases with increase in concentration in comparison with the control (Figure 3). Isoproterenol resulted in significant lipolytic activity compared to the control (*p* < 0.01).

### 3.4. Effect of* S. italica* Acetone Leaf Extract on Adipogenesis

**Figure 4:**
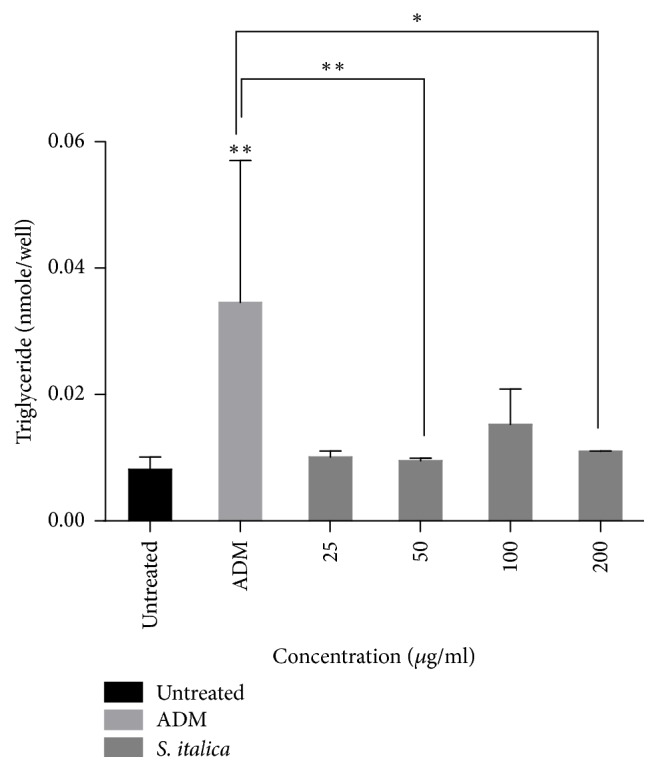
Effect of* S. italica* acetone leaf extract on adipogenesis. Cells were treated with various concentrations (25–200 *μ*g/ml) of* S. italica*. The amount of accumulated triglycerides was extracted and quantified. Adipocyte differentiation medium was used as a positive control. The experiment was carried out for 48 h. Data represents the ± SEM of 3 independent replicate experiments, ^*∗∗*^*p* < 0.01 and ^*∗*^*p* < 0.05.

Figure 4 shows the effect of the extract on adipogenesis. The extract at 100 *μ*g/ml compared to other concentrations exhibits adipogenesis activity. The positive control (ADM) significantly increase adipogenesis compared to all treatment groups.

### 3.5. Effect of* S. italica* Acetone Leaf Extract on Expression Levels of Obesity Related Adipokines

**Figure 5:**
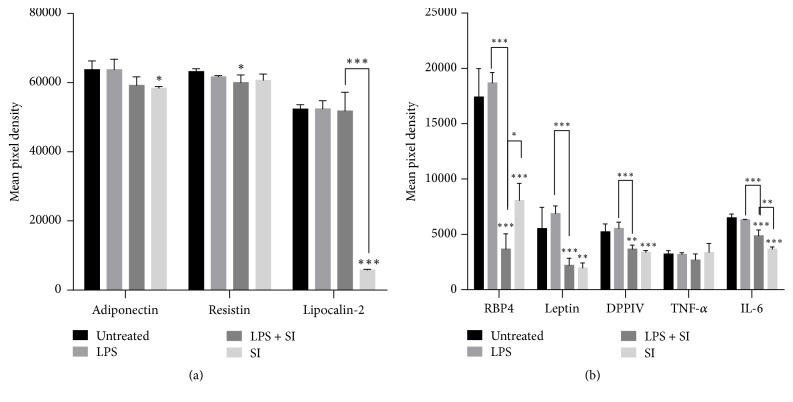
Effect of* S. italica* on the protein expression of obesity-associated adipokines. 3T3-L1 adipocytes were untreated or treated with 100 ng/ml LPS, 100 *μ*g/ml SI, and LPS in combination with SI for 24 h. Untreated cells were considered as standard control and LPS as a positive control. Data represents the ± SEM of 3 independent replicate experiments, ^*∗∗∗*^*p* < 0.001, ^*∗∗*^*p* < 0.001, and ^*∗*^*p* < 0.05.

Results show no significant effect on the expression levels of adiponectin, resistin, and lipocalin-2 after treatment with LPS. The extract shows a slight and significant decrease in expression level of adiponectin and lipocalin-2, respectively, in adipocytes that were not treated with LPS (Figure 5(a)).

Figure 5(b) shows no significant effect on the expression levels of leptin, RBP4, DPPIV, TNF-*α*, and IL-6 after treatment with LPS. The plant extract alone on the other hand shows a significant decrease in the expression levels of the above-mentioned proteins in both LPS treated and LPS untreated adipocytes except for that of TNF-*α*.

### 3.6. Effect of* S. italica* Acetone Leaf Extract on Glucose Uptake

**Figure 6:**
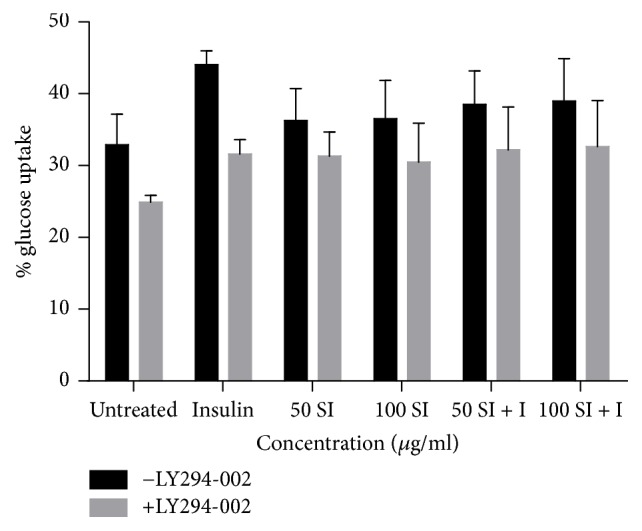
Effect of* S. italica* acetone leaf extract on glucose uptake in 3T3-L1 adipocytes. Cells were treated with various concentrations (50–100 *μ*g/ml) in the absence and presence of insulin for 4 h. LY294-002 was used to inhibit PI3K. Data represents the ± SEM of 3 independent replicate experiments.

Glucose uptake analysis of 3T3-L1 adipocytes following treatment with 50 and 100 *μ*g/ml of* S. italica* acetone leaf extract in the presence and absence of insulin for 4 h was evaluated. In order to identify the pathway through which* S. italica* stimulate glucose uptake, 3T3-L1 were pretreated with PI3K inhibitor (LY294-002). The extract shows the ability of inducing glucose uptake although there was no significant difference as compared to the control. The inhibitor at 50 *μ*M shows a decrease in insulin stimulated glucose uptake (Figure 6).

### 3.7. Effect of* S. italica* Acetone Leaf Extract on GLUT4 Translocation

**Figure 7:**
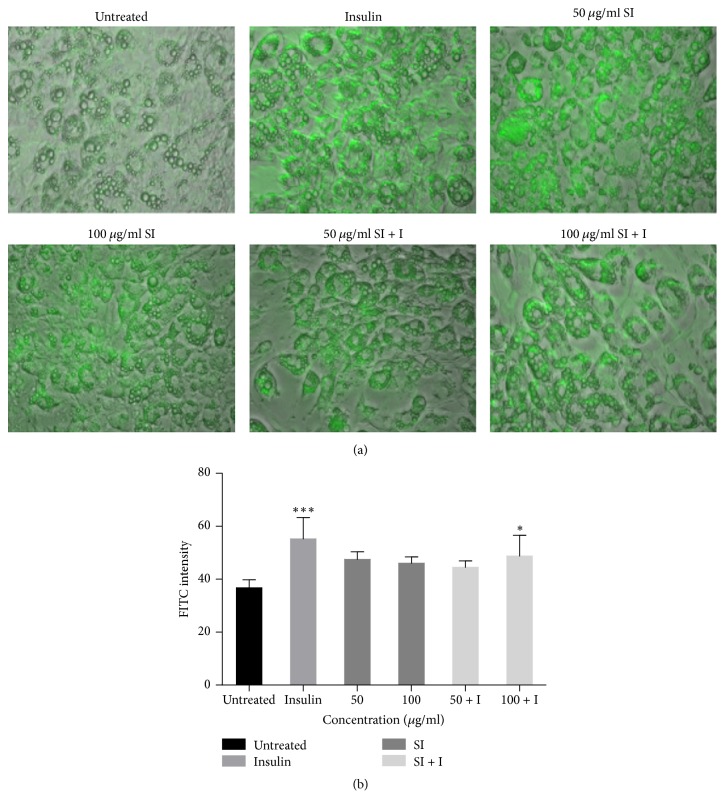
Effect of* S. italica* on translocation of GLUT4 to the plasma membrane of differentiated 3T3-L1 adipocytes. The cells were treated with 50 *μ*g/ml and 100 *μ*g/ml of the plant extract in the presence or absence of 1 *μ*M insulin and viewed with fluorescence microscope (40x magnification) (a) and quantitative immunofluorescence was measured (b). Data represents the ± SEM of 3 independent replicate experiments, ^*∗∗∗*^*p* < 0.001 and ^*∗*^*p* < 0.05.

The potential effects of different concentrations of acetone leaf extracts on translocation of GLUT4 to the plasma membrane of 3T3-L1 adipocytes are presented in Figure 7. The extract concentrations tested show a slight increase in GLUT4 translocation. The extract at a concentration of 100 *μ*g/ml in combination with insulin shows a significant increase in GLUT4 translocation (*p* < 0.05). The green fluorescence on the cell membrane indicates that GLUT4 has been effectively translocated (Figure 7(a)) and the FITC was also quantified (Figure 7(b)).

### 3.8. Effect of* S. italica* Acetone Leaf Extract on GLUT4 Expression

**Figure 8:**
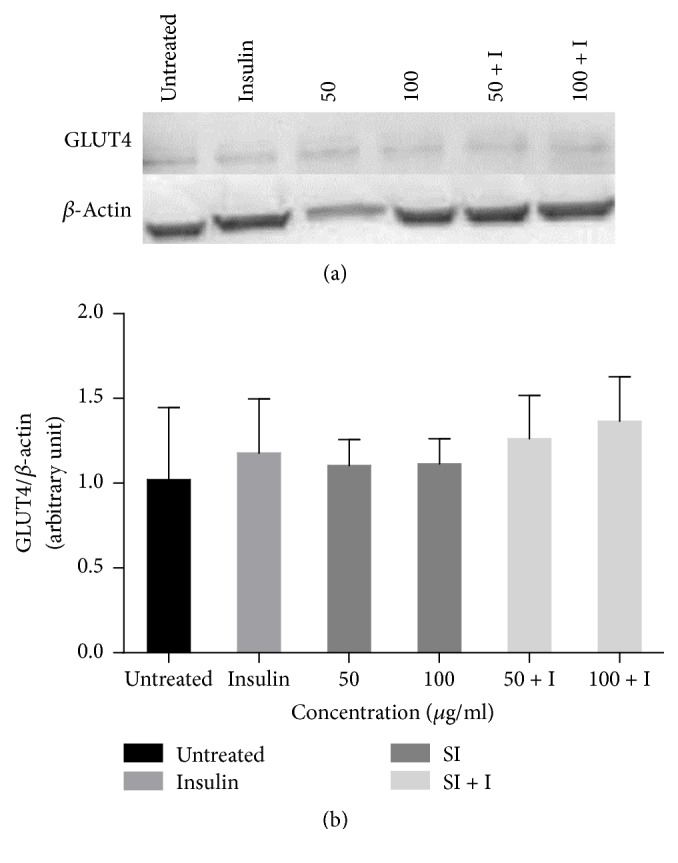
Western blot analysis of the effect of* S. italica* acetone leaf extract on the expression level of glucose transporter 4 (GLUT 4) in 3T3-L1 preadipocytes. Cells were treated with 50 *μ*g/ml and 100 *μ*g/ml of* S. italica* acetone leaf extract in the absence and presence of 1 *μ*M insulin. Data represents the ± SEM of 3 independent replicate experiments.

The cells were treated with concentrations of 50 *μ*g/ml and 100 *μ*g/ml of acetone leaf extracts of* S. italica* in the presence and absence of 1 *μ*M insulin. The extract is shown to nonsignificantly increase protein expression of GLUT4 in 3T3-L1 preadipocyte cells when in combination with insulin. Different bands in Figure 8(a) show the expression levels of different treatments and the bands were quantified (Figure 8(b)).

## 4. Discussion and Conclusion

Diabetes mellitus and obesity remain a global health problem and the use of currently available therapeutics in managing the conditions remains a huge challenge, coupled with their availability and cost to the rural poor in developing countries [15, 16]. Glucose transporter 4 (GLUT4) plays a crucial role in maintaining whole body glucose homeostasis. This is achieved through translocation of GLUT4 from an intracellular pool to the cell surface [17]. Hence, compounds facilitating GLUT4 translocation can be potentially beneficial for the treatment of diabetes [18]. Furthermore, altered balance between energy intake and expenditure can result in excess storage of triglycerides. Research in the identification of medicinal plants with potent antidiabetic and antiobesity agents with less or no side effects and readily available remains less explored [19]. The current study was aimed at determining the antidiabetic and antiobesity potential of* S. italica* through its effect on GLUT4 translocation, expression, and adipogenesis in 3T3-L1 adipocytes.

Toxicity testing plays an essential role in determining the toxic effect and characterisation of test substance. It is thus important in the development of new drugs and for the improvement of therapeutic potential of existing molecules [20, 21]. In this study, the cytotoxic effect of* S. italica* acetone leaf extract on 3T3-L1 preadipocytes was assessed. The cells were exposed to the extract for 24 and 48 h and the results show that the extract was not toxic to 3T3-L1 preadipocytes at both time intervals. The higher the CC_50_ value, the higher the concentration that is needed to kill 50% of the cells. The CC_50_ values were shown to be >1000 *μ*g/ml at both time intervals which suggests that the plant extract is not toxic. This finding could validate the safety use of the plant in clinical settings.

Glycation is identified as a key molecular mechanism of chronic diabetic complications. Identification of medicinal plants with protein glycation inhibitory potential will enhance the therapeutic strategies to delay or inhibit diabetic complications with minimum side effects [8]. Reference [22] has shown that BSA glycation is much faster in the presence of fructose. The observed decrease in fluorescence intensity during coincubation with the plant extract might suggest that the extract contains compounds that prevent or decrease the formation of advanced glycation end products (AGEs). Although there are no studies that have been reported on the antiglycation potential of* S. italica*, other authors in another study have also shown the effect of* Murraya koenigii* inhibition of glycation to be greater than that of aminoguanidine (AG), which was used as the standard inhibitor [8]. According to [23] polyphenolic compound in plants may play a major role in the inhibition AGEs. Resveratrol which has been reported to be present in* S. italica* extract [24] might have played a role in the ability of the extract to inhibit protein glycation. Recent studies have also revealed that resveratrol has beneficial effects on the liver by extenuating oxidative stress and downregulation of receptor for AGE (RAGE) expression in the liver of rats with type 2 diabetes [25].

Dysregulation of lipid metabolism is a key feature of some pathological conditions including diabetes mellitus, insulin resistance, and obesity [19]. In this study, the effect of* S. italica* on adipogenesis and lipolysis in mouse 3T3-L1 adipocyte cells was also evaluated. The lipid accumulation was measured based on the triglycerides content of the cells differentiated at various conditions. Furthermore, lipolysis was assessed through the measurements of glycerol released in the culture medium.

The current findings revealed the extract does not bring about a significant increase in adipogenesis and lipolysis when compared to the untreated control. Triglycerides accumulation during treatment with the extract was significantly lower than that of adipocyte differentiation medium which was used as a positive control for adipogenesis. Furthermore, glycerol released during treatment with the extract was also significantly lower than that of isoproterenol which was used as a positive control for lipolysis. Since resveratrol has been isolated from* S. italica* [24] and other authors [26] have observed low triacylglycerol accumulation in 3T3-L1 adipocytes treated with 100 *μ*M of resveratrol for 12, 24, and 48 h, the possibility that the presence of this compound in the extract that is responsible for the minimal induction of lipolysis in this study is high. A way of evading this is to isolate the pure compound in this extract that elicits the desired function which may not have negative effect on lipolysis. Some authors have also found resveratrol to reduce lipid accumulation in 3T3-L1 preadipocytes in a dose-dependent manner [27].

Furthermore, adipose tissues have a major endocrine function secreting multiple adipokines; many of the adipokines are involved in energy homeostasis. In obese state, adipocytes are integral to the development of obesity-induced inflammation by increasing secretion of various adipokines (including leptin, resistin, lipocalin-2, RBP4, IL-6, TNF-*α*, and DPPIV). On the other hand, adiponectin has been found to be lower in obese individuals [4]. Hence, the effect of* S. italica* acetone leaf extract on the expression levels of obesity-associated adipokines became pertinent and was thus evaluated. The results show that* S. italica* acetone leaf extract significantly decreased the expression levels of leptin, lipocalin-2, RBP4, IL-6, and DPPIV. Decrease in the expression levels of these adipokines can play a vital role in the management of insulin resistance, since these molecules are modulators of insulin resistance. However, the extract did not show any significant effect on the expression levels of resistin and TNF-*α* but rather a slight decrease which may serve as a positive indicator in the management of obesity. Furthermore, the extract is shown to slightly decrease the expression level of adiponectin which is known to be one of the adipokines that produces insulin sensitising effect. Based on the current findings, it can be suggested that the pure compound(s) from this extract that elicit the desired functions that may not negatively affect the expression level of adiponectin could be isolated.

Glucose homeostasis is determined by glucose production and utilisation in insulin sensitive organs and tissues, including muscle, liver, and adipose tissue. Glucose uptake in adipose tissues plays a fundamental role in the body glucose control [14]. Decrease in intracellular pool of transporters results from insulin resistance in peripheral tissues at the molecular level which is connected with defects in the glucose transport system. As a result, fewer transporter molecules are available for translocation to the plasma membrane in response to insulin, thus affecting the process of glucose uptake and leading to insulin resistance [28]. Thus, substances that escalate the peripheral sensitivity to insulin are useful in the treatment of type II diabetes mellitus. A previous study from our laboratory (unpublished data) has shown that* S. italica* acetone leaf extract exhibits potential in inducing glucose uptake in C2C12 muscle cells. In the present study, the effect of the extract in stimulating glucose uptake in 3T3-L1 and its possible mechanism of action were evaluated through the inhibition of PI3K.

The results showed a slight ability of the extract to induce glucose uptake in 3T3-L1 adipocytes, although the observed activity was not significant. A nonsignificant antagonistic effect was also observed when the effect of the extract was evaluated in combination with insulin, suggestive that the extract may contain compound(s) that interfere with insulin action in 3T3-L1 adipocytes. Previous findings in our laboratory showed that the extract exhibits high glucose uptake when in combination with insulin compared to insulin alone in C2C12 muscle cells, possibly due to the fact that different cell lines respond differently through different mechanisms (unpublished data). For decades, muscles have been considered to be the main site of insulin stimulated glucose uptake, with adipose tissue contributing relatively little to total body glucose disposal [29]. Reference [30] has reported that* berberine* stimulates glucose uptake in 3T3-L1 adipocytes and L6 myoblasts in an AMPK dependent manner. In this study the results showed that, during the inhibition of PI3K pathway, the glucose uptake decreased in all treatments, suggestive of its involvement in the extract-induced glucose uptake.

The translocation of GLUT4 occurs from intracellular storage sites to the plasma membrane in response to insulin. At the cell surface, GLUT4 facilitates the passive transport of glucose into muscle and adipose tissue [31]. Increased GLUT4 expression on the plasma membrane is important for the uptake of glucose into the adipose and muscle tissues and plays a key role in maintaining normal blood glucose level [32].

The current findings showed* S. italica* acetone leaf extract to have increased GLUT4 translocation, even though the increase was not as significant when compared to the positive control. Cotreatment with insulin showed a nonsignificant antagonistic effect, again suggesting the presence of substances within the extract that interfere with insulin activity. The AKT pathway has been reported to increase translocation of GLUT4 to the plasma membrane in muscle and adipose tissues [33]. In another study, [34] attributed resveratrol-mediated activation of AKT in their study to have played a vital role in GLUT4 translocation. Since resveratrol has been isolated from* S. italica*, the possibility of its involvement in facilitating glucose uptake in this study is paramount. Findings by [32] demonstrated that gallic acid induced GLUT4 translocation through the PI3K pathway in 3T3-L1 adipocytes. It is highly likely that* S. italica* might also be inducing GLUT4 translocation through a similar pathway, in this instance. This might be confirmed by the inhibitory effect of LY294-002 on* S. italica*-induced glucose uptake.

The expression of GLUT4 levels in treated cells was also assessed. GLUT4 is selectively expressed in insulin responsive tissues such as adipose and skeletal muscle cells [14]. According to [35], GLUT4 is not highly expressed in fibroblastic state. In this study 3T3-L1 preadipocytes were used to determine the effect of* S. italica* on GLUT4 expression. The results show that* S. italica* extract increased GLUT4 expression, as evidenced by the increase in GLUT4 protein band density following treatment with the extract (Figure 8(a)). Increase in GLUT4 expression might be due to the presence of flavonoids in the extract. Several studies have suggested the important role of flavonoids in enhancing the expression of GLUT4 [36]. The extract in combination with insulin resulted in higher GLUT4 expression. This shows a synergy between the extract and insulin in inducing GLUT4 expression levels.

In conclusion, the results suggest that* S. italica* stimulates glucose uptake through the PI3K-dependent pathway. The extract might play a role as a therapeutic agent for obesity through its ability to downregulate some of obesity-associated adipokines. Furthermore, the extract might have compounds that have antiglycation capabilities. However, further studies are necessary in order to understand which compound(s) is/are responsible for the regulation of obesity-associated adipokines.* In vivo* studies are worth undertaking in order to determine the effect of* S. italica* extract within the living system.
